# Neurotrophic and Neuroregenerative Effects of GH/IGF1

**DOI:** 10.3390/ijms18112441

**Published:** 2017-11-17

**Authors:** Vittorio Emanuele Bianchi, Vittorio Locatelli, Laura Rizzi

**Affiliations:** 1 Endocrinology and Metabolism, Clinical Center Stella Maris, Strada Rovereta, 42-47891 Falciano, San Marino; 2 School of Medicine and Surgery, University of Milano-Bicocca via Cadore, 48-20900 Monza Brianza, Italy; vittorio.locatelli@unimib.it; 3 Molecular Biology, School of Medicine and Surgery, University of Milano-Bicocca, via Cadore, 48-20900 Monza Brianza, Italy; laura.rizzi@unimib.it

**Keywords:** growth hormone, IGF-1, neuroregeneration, amyotrophic lateral sclerosis, Alzheimer’s disease, peripheral nerve regeneration, testosterone, 17β-estradiol

## Abstract

Introduction. Human neurodegenerative diseases increase progressively with age and present a high social and economic burden. Growth hormone (GH) and insulin-like growth factor-1 (IGF-1) are both growth factors exerting trophic effects on neuronal regeneration in the central nervous system (CNS) and peripheral nervous system (PNS). GH and IGF-1 stimulate protein synthesis in neurons, glia, oligodendrocytes, and Schwann cells, and favor neuronal survival, inhibiting apoptosis. This study aims to evaluate the effect of GH and IGF-1 on neurons, and their possible therapeutic clinical applications on neuron regeneration in human subjects. Methods. In the literature, we searched the clinical trials and followed up studies in humans, which have evaluated the effect of GH/IGF-1 on CNS and PNS. The following keywords have been used: “GH/IGF-1” associated with “neuroregeneration”, “amyotrophic lateral sclerosis”, “Alzheimer disease”, “Parkinson’s disease”, “brain”, and “neuron”. Results. Of the retrieved articles, we found nine articles about the effect of GH in healthy patients who suffered from traumatic brain injury (TBI), and six studies (four using IGF-1 and two GH therapy) in patients with amyotrophic lateral sclerosis (ALS). The administration of GH in patients after TBI showed a significantly positive recovery of brain and mental function. Treatment with GH and IGF-1 therapy in ALS produced contradictory results. Conclusions. Although strong findings have shown the positive effects of GH/IGF-1 administration on neuroregeneration in animal models, a very limited number of clinical studies have been conducted in humans. GH/IGF-1 therapy had different effects in patients with TBI, evidencing a high recovery of neurons and clinical outcome, while in ALS patients, the results are contradictory. More complex clinical protocols are necessary to evaluate the effect of GH/IGF-1 efficacy in neurodegenerative diseases. It seems evident that GH and IGF-1 therapy favors the optimal recovery of neurons when a consistent residual activity is still present. Furthermore, the effect of GH/IGF-1 could be mediated by, or be overlapped with that of other hormones, such as estradiol and testosterone.

## 1. Introduction

Human neurodegenerative diseases, such as ALS [1], Alzheimer’s [2], Parkinson’s disease [3], and prion disorders, are related to the aging process, and represent a constant increasing economic and social burden for modern society. Maintenance of the efficiency of CNS is particularly important in aging and for the control of metabolism. The GH/IGF-1 axis plays the main role in brain growth, development, and myelination, but also in the neurogenesis process and plasticity [4]. It is a difficult task to distinguish between the effects of GH from IGF-1, due to the half-life of GH in plasma and to the cross-activation of receptors. Both GH and IGF-I can cross from the blood to the brain by physiological mechanisms [5,6]. IGF-1 may get in the brain parenchyma capillaries through the blood brain barrier, and they may be filtered through the choroid plexus into the cerebrospinal fluid [6]. Furthermore, IGF-1 sustains the brain through the activation of IGF-II gene expression, as well as by uptake into the cerebrospinal fluid [7]. Experimental models in animals showed that GH is absorbed via the capillary of blood brain barrier, supporting the concept that GH influx happens by simple diffusion, although a specific transport system has not been demonstrated [5].

GH/IGF-1 are determinant regulators of cellular function, and an impaired release of GH and IGF-1 with advancing age leads to severe alterations in tissue structures and functions, especially within the brain [8]. GH is secreted by the anterior pituitary gland, and the primary effect is the activation of GH receptors and the secretion of IGF-1, mainly by the liver [9], and locally by the brain [10]. The effects of GH are mediated by the transmembrane GH receptors, which are expressed on the surface of most cells [11]. Therefore, GH acts through two independent mechanisms of action: one activating the cellular GH receptors, and the other inducing the IGF1 secretion by the liver. Insulin-like growth factors (IGFs) are transported in the blood by six binding proteins, IGF-BP1–6, and IGF-1 is transported mainly by IGF-BP3 [12]. At the CNS level, a high expression of GH and IGF-1 receptors has been shown [13,14], suggesting that brain cells, such as neurons, glia, and oligodendrocytes, actively respond to GH and IGF-1 signaling. Pulses of GH and IGF-1 increased significantly only around adolescence, and the amplitude of GH releases increases the level of circulating IGF-1. The decline of GH secretion [15] results in a decrease in circulating IGF-1 levels [16]. IGF-1 can also be synthesized within the cerebral and peripheral nerve cells, with the purpose of stimulating the development and growth of neurons and glial cells [17].

GH can act directly on the brain, by activating GH receptors located on the membrane of the cerebral cortex neurons, on neurons of the thalamus and hypothalamus, on Purkinje cells of the cerebellum, on neurons of the trapezoid body of the brainstem, and on retinal ganglion cells [14]. In embryonic cultures from cerebral cortex, it was demonstrated that GH stimulated neuronal precursor and glial cells [18,19], increased neurogenesis, myelination, and synaptogenesis, [4]. These effects of GH are mediated by the local production of IGF-1 and IGF-BP3 [18]. In adults, GH administration regulates brain function, learning, memory, and neuroprotection [20].

Glial cells, including microglia, astrocytes, and oligodendrocyte lineage cells, are also strongly reactive to GH [14,21]. GH deficiency in mouse causes the reduction in oligodendroglial cells and the myelination process [22]. Astrocytes, which are the most abundant and well-studied type of glial cell in the adult brain [23], play a crucial role in the maintenance of brain health, protecting it by inflammation [24]. GH, when administered intravenous (iv), exerts its effect on astrocytes, and seems to mediated by the PI3K/Akt pathway [25]. Oligodendrocytes are responsible for the myelin production in axons, and gives them trophic support, ensuring long-term integrity [26]. Loss of myelin is responsible of various neurological diseases, and contributes to neuropsychiatric disorders.

Glial cells are involved in providing neurotrophic signals to neurons required for their survival, proliferation, and differentiation. Furthermore, it is recognized that glial cells have some effects on certain physiological processes, such as breathing, and in assisting neurons to form synaptic connections amongst one another [27]. Astrocytes maintain the homeostatic environment of the CNS and play an important role in immune regulation, acting a source of chemokines, cytokines, and effector molecules [28]. Growth factors (GFs) typically act as signaling molecules between cells [29]. They perform an essential function in the route of signaling molecules between astrocytes and neurons [30].

GH-induced neuroprotection downregulating the apoptosis-promoting gene [31] protected the brain from hypoxic injury, attenuating caspase-3 expression [32]. These data indicated that exogenous GH therapy might exert a protective action against hypoxic-ischemic brain injury, and also promotes the proliferation of progenitor neural stem cells in the human fetal cortex [33]. Interestingly, GH affects most of the major brain neurotransmitters, including the monoamine (such as serotonin and noradrenaline) [34], the dopaminergic system [35], and stimulates dopaminergic activity [36].

IGF-1 has a more potent trophic effect, compared to GH, in the motor and sensory neurons, and on neuronal development and regeneration [37,38,39,40,41]. A reduced IGF-1 signaling, due to mutation of IGF-1 and its receptor gene, caused microcephaly and mental impairment [42,43]. IGF-1 promotes neurite outgrowth [17] and protects cells from apoptotic stimuli at mitochondrial level [44]. Furthermore, IGF-1 is active in Schwann cells’ survival, maturation, and myelination in vitro [40,45]. In denervated muscle tissue, IGF-1 stimulates muscle growth and regeneration, and prevents atrophy [46,47,48]. The bioavailability of IGF-1 and IGF-2 and their binding proteins (IGF-BP2 and IGF-BP3) could play an important role in the prevention and treatment of several neurologic disorders [49]. Emerging promising results demonstrates that IGF-1 possesses a therapeutic effect on the brain by increasing hippocampal neurogenesis and memory accuracy in elderly individuals, and possibly, in neurodegenerative disorders [50]. Mashayekhi et al. [51] found the serum level of IGF-1 and IGFBPs were higher in patients affected by Parkinson disease compared to controls, but the significance of these data in the pathophysiology of the disease remains unexplained. Vincent et al. [52] showed that IGF-I administration determined a protective effect, dose-and time-dependent, on neurons acting on both the MAPK, a well-known signal transduction pathways marker [53], and PI-3K/Akt pathways, promoting the survival of motor neurons.

For these reasons, GH and IGF-1 could represent a novel strategy therapy in neurodegenerative diseases such as ALS, Alzheimer’s disease (AD), Parkinson, and dementia. An increasing amount of evidence is suggesting the potential usefulness of growth factors, such as IGF-I, as potential treatments for certain neurodegenerative diseases, including ALS and AD due to its neurotrophic effect [54]. Data about the effects of GH and IGF-1 administration in neurodegenerative diseases are still insufficient. This study aims to evaluate the effects of GH and IGF-1 therapy conducted in human and animal models on the peripheral and central nervous system, and future perspectives.

## 2. Methods

A systematic literature search was performed using PubMed Medline, Google Scholar, and Cochrane Central Register of Controlled Trials. Between these search engines, Google Scholar provided a large amount of data, but is less selective. A combination of the following keywords was used: “GH”, “IGF-1” associated with “neuroregeneration”, “Amyotrophic Lateral Sclerosis”, “Alzheimer’s disease”, “brain”, “Parkinson’s disease”, “neuron.” Clinical trials have been selected. AD and PNS regeneration pre-clinical studies were also reviewed. No exclusion criteria were adopted, due to the limited number of clinical trials found.

We have searched separately for “GH” and “ALS”, “GH” and “Amyotrophic lateral sclerosis” and obtained different results (the first search gave more than double than last one). The different results were obtained in PubMed, Google Scholar, and Cochrane. Searching “IGF-1 administration”, “neurodegenerative disease” in PubMed fetched 107 articles. Including the filter “clinical trials” and “humans”, we obtained 11 articles. Of these, only 2 have been selected. Searching for “IGF-1 administration” and “neuron” of 264 articles, after filters, we obtained 3 articles and only 1 was selected. “IGF-1 administration” with “ALS” found 70 items, 24 were selected, and 2 were useful. Using the words “IGF-1 therapy” with “ALS” of 158 items, 40 were selected, and 7 useful. In Cochrane library, for the same items, we found only 3 studies, and selected 2. Similar results were found for “IGF-1” and “Amyotrophic Lateral Sclerosis”. No study matched for GH and IGF1 therapy for Huntington’s Disease.

## 3. Results

**Figure 1:**
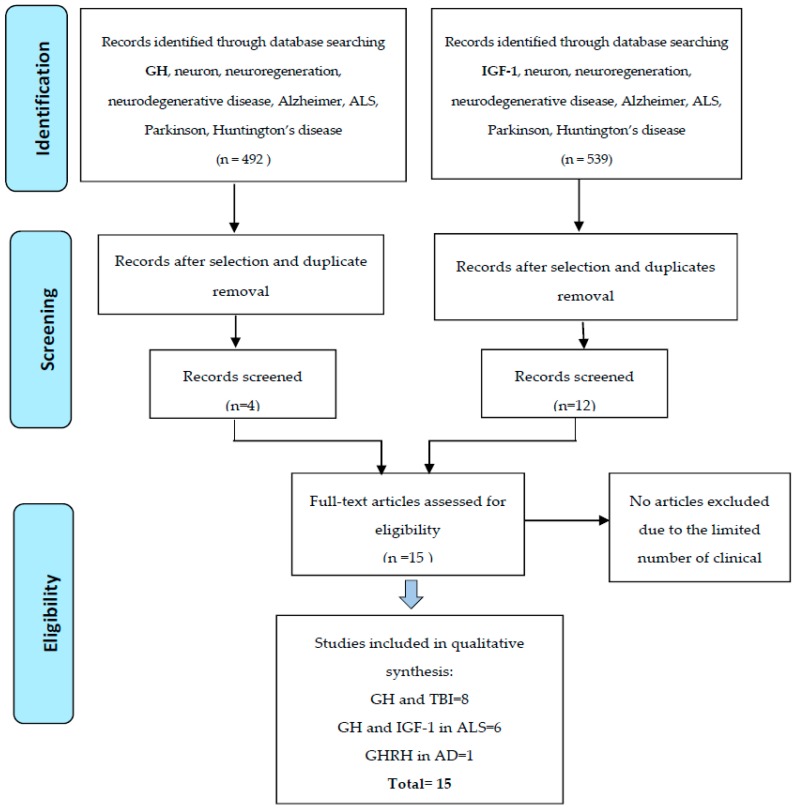
Flow chart illustrating the literature search and selection.

Of the retrieved articles, we selected eight articles on the effect of GH in patients who suffered from traumatic brain injury (TBI), enrolling 1140 patients with a mean age of 41.0 ± 10.6 (Table 1). Six studies on the effect of GH and IGF-1 in patients with ALS, enrolling 741 patients with a mean age of 53.2 ± 5.42 years (Table 2). One study evaluated the effect of GHRH (growth hormone releasing hormone) in patients with AD. The neuroregenerative effect of GH and IGF1 on peripheral nerve regeneration (PNF) has been considered, although no clinical studies in human have been found. While the administration of GH in patients after TBI showed a significantly positive recovery of brain and mental function, IGF-1and GH therapy in ALS evidenced some contradictory results. The data are summarized in the flow chart (see Figure 1).

### 3.1. Mechanisms of Action

IGF-1 has a high affinity for its receptor, a tyrosine kinase receptor which mediates PI3K-AKT. Akt is a critical mediator of growth factor-induced neuronal survival [55]. Brain IGF-1 signaling has a major inhibitor of GSK3β, activating anabolic and neuroprotective effects [56]. The MAP kinase pathways play a fundamental role in neuronal tropism and survival, and prevent neuronal apoptosis [57,58]. Additional pathways linked to IGF-I signaling include the JNK, p38 MAPK, and mTOR signaling pathways. The mitogen-activated protein kinase (MAP-kinase) pathway is activated by many extracellular stimuli, and exerts a fundamental role in human pathology. Its inhibition (obtained by removing particular JNK genes) reduces the severity of various diseases, including Parkinson’s disease and cerebral ischemia [59]. Inhibitors of JNK have been used as a therapeutic strategy for neuroprotection in the retina [60], and multitargeting inhibitor protein kinases have been proposed as the most promising strategy to treat Alzheimer’s disease [61]. The growth factors that have been demonstrated to participate in motor neuron physiology are vascular endothelial growth factor (VEGF), glial-derived neurotrophic factor (GDNF), ciliary neurotrophic factor (CNTF), and IGF-1. Trophic factors exert their mechanism of action, stimulating the motor neuron function and survival, and may have a potential therapeutic activity on clinical outcome of various neurologic disorders [62]. High IGF-1 level protects against β-amyloid deposition, and the insulin/IGF-1 signaling pathway shows a neuroprotective action [63]. The infusion of IGF-1 in the brain of old rats cleared the β-amyloid deposition reaching the level of young animals [64], while the reduction of serum IGF-1 level in AD animal model accelerated the deposition of β-amyloid plaques in the brain [65]. Insulin is the main stimulator of β-amyloid release by neurons [66], also observed in older subjects [67]. However, IGF-1 activating insulin receptors can stimulate β-amyloid deposition also. Insulin/IGF-1 signaling is extremely important in the control of neurogenesis [68], because it can regulate neural, stem cell proliferation, differentiation, and survival [69]. Astrocytes have a different susceptibility compared with neurons to insulin/IGF-1 activity. Insulin receptor in the nucleus has a variable range of expression at a molecular level, suggesting that the signaling is not limited to the cytoplasm [70].

A reduced insulin/IGF-1 signaling in experimental animal model protects the mammalian brain from amyloid-β toxic effects, neurodegeneration, and Alzheimer disease [71]. Similar results have been obtained by providing adenoviral administration of IGF-1 [72], and reducing IGF-1 signaling [73]. However, whether IGF-1 signaling is protecting the mammalian brain is still under discussion. Reducing insulin/IGF-1 signaling alters the aging process [36,44], and the major risk factor for the development of neurodegeneration is aging [74]. Thus, reducing rather than increasing IGF-1 signaling should ameliorate aging-associated diseases.

Gontier et al. [71] demonstrated in a mouse model that the progression of the AD is significantly delayed when IGF signaling is blocked. In experimental AD animal models, a less abundant deposition of β-amyloid and reduction of neuroinflammation was observed. Also, increased β-amyloid load and plaque formation were considered, in many studies, as a marker of toxicity [65,75]. A conflict arises because reduced insulin/IGF-1 signaling in the CNS is associated with longevity, but can dysregulate glucose and energy homeostasis, and promote being overweight. White et al. [76] have explored how the genetic manipulation of insulin/IGF-1 signaling system could influence systemic metabolism, lifespan, and neurodegeneration. In the complex mechanism of insulin/IGF-1 signaling on the neuron, the interaction with other factors, such as sex hormones and nutrition, to regulate nutrient homeostasis, should be considered [69,77]. The insulin/IGF-1 signaling effect on neuron and glial cells shows somewhat contadictory aspects, not yet completely explained. However, it should be considered that the effects of insulin/IGF-1 signaling have different actions on healthy neurons and astrocytes, with respect to sick cells.

### 3.2. GH Therapy in Traumatic Brain Injury (TBI)

Head injury of moderate or severe intensity determines a condition of hypopituitarism in approximately 40% of patients [78], with a consequent neuroendocrine disorder [79]. Three months after a TBI, hypopituitarism is still evident in 54–56% the patients [80], and persisted in 36% of the patients after one year, involving the gonadotropic axis in 21%, the somatotropic axis in 10%, and the corticotropic axis [81]. We have selected nine clinical trials, which evaluated the effect of GH administration in patients after TBI, enrolling 1180 patients with a mean age of 44.2 ± 9.5. In all studies, a significant improvement of the quality of life, memory, cognition, and motor control have been shown [82,83,84,85,86,87,88,89,90]. The studies are summarized in Table 1.

KIMS database, from a primary care cohort in Germany, the Kabi International Metabolic Database (KIMS), includes a healthy sex- and age-matched control group [88,91]. The doses of GH administered varied from 0.2 up to 0.6 mg/day, and the duration of therapy was mainly one year; in one study, three months [85]; in another, six months [87]. One study evaluated the effect of a very short-term treatment (14 days) with intravenous infusion of GH or IGF-1 at doses of administration of 0.05 mg/kg/day for GH, and 0.01 mg/kg/h for IGF-1 [89]. In the report of Devesa et al. [90], only 5 out of 13 patients were GH deficient, but positive results were obtained in both types of patients. The beneficial effect of GH administration potentiates the effect of GH locally produced to stimulate neural repair [92]. Dysartia, dysphonia, and tongue paralysis disappeared, while blindness persisted in some cases [90]. Combined GH plus IGF-1 administration in TBI patients showed more consistent improvements [89]. The treatment was well tolerated in all patients. In conclusion, from these studies, it appears that in patients with TBI and also short time therapy with GH, from 14 days to three months, a low dose of 1 mg/day is effective in determining brain recovery. The decision to make a treatment with GH to promote the recovery of brain function, and the improvement of the clinical outcomes should be considered [93]. Considering the low-risk level of the therapy, the limited number of clinical trials found in the literature shows that clinicians have not yet implemented the utility of GH therapy in such patients.

### 3.3. GH/IGF-1 Treatment in Amyotrophic Lateral Sclerosis (ALS)

ALS is a disease of the nervous system, characterized by degeneration of both upper and lower motor neurons of the spinal cord, brainstem, and cortex [94]. The disease causes frontotemporal dementia, progressive muscle and strength loss, dysphagia, dyspnea, and progressively, death [95]. ALS and frontotemporal dementia are different diseases with similar clinical and pathological conditions [96], but the pathogenesis of motoneuron degeneration is not yet entirely understood. ALS has familial transmission of 20–25%, with clinical evolution similar to sporadic ALS [97]. Swedish and UK studies evaluated the incidence of heritability of 61% [98]. ALS was characterized by mutations in the gene for superoxide dismutase-1 (SOD1) [99], with an incidence of 10–20% of ALS patients and 1–2% in sporadic ALS [100]. More than 100 type SOD1 mutations that cause ALS are known [97]. Steyn et al. [101] found in male mice, genetically hSOD1(G93A), had a reduced pulsatile secretion of GH and a decreased plasma IGF-1 level, and reduced IGF-1 receptor expression at muscular and lumbar spinal cord levels. Interestingly, the alterations in GH and IGF-1 secretions in these mice have been found in 50–70% of human patients with ALS [102,103]. These data demonstrated a correlation between GH deficiency and hSOD1(G93A) expression, and are essential for the consideration of GH and IGF-1 therapy in ALS.

Other proposed mechanisms in ALS pathogenesis are glutamate-induced neurotoxicity with increased glutamate concentration in cerebrospinal fluid [104], and altered oxidative stress reactions, protein and DNA damage, alterations in axonal transport, and dysregulated autophagy [105]. Saenger et al. [106] investigated the effect of IGF-1 in two SOD1-G93A mouse lines that express a pathology similar to familial ALS (the milder and the more severe phenotype form). Results showed that in a milder form, pegylated-IGF-1 treatment, significantly improved muscle force, motor coordination, and animal survival. In contrast, treatment of more severe form with pegylated-IGF-1 or IGF-1, even at high doses, did not increase survival or functional outcomes, despite increased signaling in the brain and spinal cord by both agents. These findings show that IGF-1 treatment can be effective only in milder forms of ALS, or when the efficiency of the neuron is still preserved. The functional improvement induced by IGF-1 treatment is dependent on the severity of disease [106].

The protective effect of GH in familial ALS has been demonstrated in both in vitro and in vivo models [107]. In patients with ALS, a significant reduction of GH secretion was found with higer incidence in male than in women, 83% versus 60%, respectively [102]. The plasma IGF-1 level is also reduced, suggesting an involvement of the GH/IGF-I axis in the development of ALS [108]. Only two studies evaluated the effect of GH therapy in ALS human subjects. Smith et al. [109] found that in 75 patients with ALS, that 18 month GH administration did not determine any clinical improvement in the treatment group, compared with controls. The poor prognosis was more severe in patients with early loss of muscular strength, indicating that GH therapy had been ineffective on motor neuron function. Similar results were found by Saccà et al. [84], that demonstrated no beneficial effect on neuronal loss, and motor function in patients with ALS treated with GH. The evaluation of the upper motoneuron loss had been determined with magnetic resonance spectroscopy. Kaspar et al. [110] showed that GH therapy associated with exercise might exert a synergistic effect. Lunetta et al. [111] described motor neuron degeneration in ALS disease due to an abnormal expression of IGF-1 in the skeletal muscle, suggesting that IGF-1 expression in muscular tissue is essential to maintain the motor neuron efficiency. In our search, we retrieved only four clinical studies, reporting the effect of IGF-1 administration in ALS [112,113,114,115], and two on GH effects in ALS [84,109], see Table 2.

Lai et al. [115] published a study conducted in 266 patients, treated in the USA with two different doses (0.05 and 0.10 mg/kg/day) by subcutaneous administration of rhIGF-I. The treatment led to a 26% deceleration in functional impairment compared to placebo-treated patients. High and low doses produced similar effects. Borasio et al. [114] conducted a similar placebo-controlled trial study in 183 ALS patients, from 8 European countries, receiving for nine months, rhIGF-1, 0.10 mg/kg/day subcutaneously, and did not find any significant difference among groups. Both studies used a similar dose of therapy and endpoints (disease progression assessed by the Appel ALS rating scale), and found that rhIGF-I was safe and well-tolerated. The analysis of the data derived from both studies showed a significant benefit to high-dose rhIGF-I treatment on the primary clinical outcome [116]. No data on survival endpoint was available. Nagano et al. [113] found that high doses of IGF-1 (3 μg/kg body weight) slowed the decline of motor functions of the ALS patients. In the study of Sorenson et al. [112], a high incidence of deaths (163 patients, 51%) was observed in the course of the study. Among these, 104 deaths occurred in patients during treatment, and 53 occurred in patients who had discontinued their treatment before death. Twelve patients died within one month of completing their 24 months of treatment. The most common adverse effect described was localized reaction at the site of injection. There were 34 thromboembolic events, but the incidence of thromboembolic events in patients with ALS was similar to that observed in other studies, and not dependent on the treatment [117]. No benefit in the patients under treatment was observed. The mortality rate reported in this study evidenced a severe and irreversible damage to the neurons, and that any therapy could be useless. However, Howe et al. [118] suggested that the conclusion of Sorenson et al. [112] cannot be considered definitive, because the low dosage of IGF-1 administered can nullify the effect of treatment. The treatment with IGF-1 in ALS patients should consider that, when activated, the IGF-1 receptors in motor neuron can induce the formation of IGF-BP 2, 5, and 6, which determines the reduction of local free IGF-1 [119]. Various IGF-binding proteins in ALS patients are elevated, while IGF-1 circulating levels are reduced, suggesting that in these patients, the peripheral IGF-1 system plays an important role [108]. At the moment, the effects of GH and IGF-1 therapy in ALS remain uncertain. First of all, clinical trials are very limited. Only two clinical trials evaluated the effect of GH therapy in patients with ALS [84,109]. Smith et al. [109] used a very low dose (0.1 mg/day) of GH, which was ineffective, as demonstrated by the unchanged plasma level of IGF-1 after therapy. In the Saccà study [84] (8 UI = 2.8 mg/day), the IGF-1 and IGF-BP3 were found reduced after the therapy demonstrating the inefficacy of GH administered. Both studies evidenced that GH administration had been ineffective, probably due to the interference of other factors (inflammation, nutrition, physical exercise). Given that the treatment is safe, the rhIGF-I therapy in ALS should be considered useful, but more specific clinical trials are necessary, to evaluate and clarify its clinical efficacy.

### 3.4. Peripheral Nerve Regeneration

Unfortunately, there are no clinical trials conducted in humans available, and the data reported are summarizing preclinical animal studies. Overall data indicate that PNS is a target of the IGF-I/mTOR pathway that is essential for mitochondrial activity in regulating cell growth and proliferation [120]. Schwann cells are displayed along the axon and produce the myelin sheaths, which are essential for neural stimuli transmission (see Figure 1). After crush nerve lesion or interruption, Schwann cells undergo atrophy, due to the lack of contact with proximal neurons [121]. Consequences of nerve cut are the muscle atrophy and strength loss, and the duration of chronic denervation causes atrophy and fibrosis, which determine a limited recovery [122]. Chronic denervation of distal nerve after a long time severely compromises the ability of the axons to regenerate into the distal nerve stumps, and the survived Schwann cells undergoing atrophy lose their ability to myelinate the axons [123]. Peripheral nerve injuries result in debilitating motor and sensory deficits [124].

Kanje et al. [125] showed in hypophysectomized rats that, after sciatic nerve crush injury, the axonal regeneration was impaired, but was restored after GH administration. Further studies [126] demonstrated that after peripheral nerve injury in animals, GH therapy determined the muscle reinnervation and reduction of muscle loss, accelerating axonal regeneration and myelination. IGF-1 is a growth factor able to stimulate the sprouting of axons and the survival of neurons, accelerating myelination and axonal regrowth in vivo, and both proliferation and differentiation in Schwann cells [38].

By using immunohistochemical techniques in rats, the expression of IGF-1 in the nervous system has been investigated. Following crush injury of a motoneuron, accumulation of IGF-1 immunoreactive material within the damaged area was shown, in vitro [127]. The local administration of IGF-1 improves the recovery of facial nerve function after a crush injury [128]. Furthermore, gonadal hormones [129], like estradiol [130] and testosterone [131] contribute increasing axon regeneration after peripheral nerve section. Tuffaha et al. [132] demonstrated that during GH therapy in mice, the peripheral nerve injury accelerates axonal regeneration and myelination, reduces muscle atrophy, and enhances muscle reinnervation. Neuromuscular junction analysis demonstrated a significantly greater percentage of reinnervation of motor endplates in growth hormone-treated animals compared with control. In humans, due to the longer extension of the axons than in rats, the injury of the distal nerve fibers leaves the muscles without axonal contact for a greater period of time, so that therapy with GH would be essential for a rapid clinical outcome. In summary, these studies demonstrated that GH and IGF-1 have a motoneuronal trophic activity, and participate in motoneuron axonal regeneration and sprouting, indicating that they can play a major role in the stimulation of their recovery after injury [133]. In humans, strategies to increase the speed of axonal growth and improve functional outcomes, are necessary to keep target tissues receptive to reinnervation. Despite the demonstrated efficacy of GH and IGF-1 therapy on peripheral nerve injury in the experimental animals, to our knowledge, no clinical studies have been conducted in humans.

### 3.5. GH/IGF-1 and Alzheimer’s Disease (AD)

AD is a form of neurodegenerative disease accounting for 50–70% of all causes of cognitive impairment [134], and is strongly correlated to amyloid-β (Aβ) deposition, causing the neurodegeneration [135]. Many risk factors are implicated in the pathogenesis of AD, and cognitive impairment remains the primary focus of prevention [136]. A recent review on clinical studies of mild to moderate dementia cases showed no real progress in identifying disease-modifying treatments [137]. Diet and antioxidant deficiencies have been proposed [138]. However, a large population study, aimed at evaluating the effect of the supplemental use of vitamin E and selenium to prevent dementia, showed that these antioxidants did not forestall dementia, and are not recommended as preventive agents [139]. In AD pathogenesis, both estrogens and androgens have been implicated because they exert a wide range of protective actions on brain structures [140]. Low testosterone level in the brain is correlated with AD incidence, and inversely correlated with β-amyloid deposition in men [141], while in female, 17β-estradiol has a protective effect against AD [142]. In patients affected by AD, the GH/IGF-1 axis is downregulated [143], and in particular, IGF-1 therapies could provide significant benefits to these patients [144].

Inflammation plays an important role in the pathogenesis of AD, and represents the predominant mechanism of activation and progression of the disease [145]. High serum level of pro-inflammatory cytokines anticipates the detection of β-amyloid in the brain [146], suggesting that they can stimulate amyloid precursors [147] and drive AD development [148]. Inflammation in the brain occurs early, and is evident also in the preclinical condition of the AD, favouring the development of the pathology [149]. An inverse correlation between inflammatory processes with the level of sex steroids has been observed and reviewed by Uchoa et al. [150].

Friedman et al. [151] evaluated the effect of the growth hormone-releasing hormone (GHRH) analog, tesamorelin (1 mg/day, for 20 weeks), on 30 adults with a mean age ranging from 55 to 87 years. They found increased GABA levels in all three brain regions (dorsolateral frontal, posterior cingulate, and posterior parietal), increased *N*-acetylaspartylglutamate levels in the frontal cortex, and decreased myoinositol concentrations in the posterior cingulate, which are linked to the AD. The study demonstrated a favorable effect of GHRH administration in adult patients with mild cognitive impairment, independent of the IGF-1 level that did not change significantly.

The neuronal defenses against AD rely on a neuroprotective response activated by genetic disruption of IGF1R signaling. George et al. [152] evidenced the neuronal IGF1R signaling as a relevant target to prevent AD. McGinley et al. [144] generated human cortical neural stem cells that stably produced IGF-1, and were then transplanted into double-transgenic mice, a model used for AD. These novel human cortex-derived cells preferentially differentiated into gamma-aminobutyric acidergic neurons, a subtype of dysregulated neuron in AD, produced an increase of vascular endothelial growth factor levels, and display an increased neuroprotective capacity in vitro. The authors found a significantly reduced level of death. This supports the idea that IGF-1 provides an increased protection of spinal cord neural stem cells to cellular insults, and could represent a model for a future therapeutic strategy for AD. Nowadays, no clinical studies have evaluated the effect of GH/IGF-1 in the prevention and treatment of patients with AD.

## 4. Discussion

GH and IGF-1 play an important role as neurotrophic factors within the PNS and CNS [153]. In particular, IGF-1 is strongly implicated in neurogenesis, synaptogenesis [154], and exerts antiapoptotic and anti-inflammatory effects [155] at cortical, motor, and sensory levels [156]. GH and IGF-1 are largely implicated in brain repair after injury [157], and stimulate the survival of mature and immature neurons at hippocampal dentate gyrus [158,159], and promote the proliferation of neuronal precursors [160]. These assumptions implicate that GH and IGF-1 should be considered for the prevention and treatment of various neurological diseases. However, in spite of the demonstrated neurotrophic effect of GH and IGF-1 in animals, very limited clinical applications in human subjects have been reported. In patients with traumatic injury of brain neurons, as observed in TBI (Table 1), the treatment with GH showed a positive effect, probably related to the young age of the patients and healthy condition of neurons. Similarly, after traumatic, ischemic injury, as observed in the acute stage of stroke, higher IGF-1 levels correlated with a better neurological recovery and physical outcome [161,162,163]. IGF-BP3 has a prognostic and independent value to predict the functional outcome after ischemic stroke [164]. Lower plasma levels of IGF-1 predicted unfavorable functional outcomes and death [165].

GH and IGF-1 administration in patients with ALS has shown modest and contradictory results (Table 2). Only the study of Nagano et al. [113] showed a slower progression of ALS, at higher doses of intrathecal administration of IGF-1 (3 μg/kg body weight), and Lai et al. [115] at high and low doses (0.05 mg/kg/day or 0.10 mg/kg/day) in ALS patients. Other studies, as well as GH therapy, showed no positive effects [112,114].

The controversial effect of GH/IGF-1 in ALS patients it is complex to explain, and can be related to the severity of the disease with a high short-term mortality rate [112], suggesting that the recovery of neurons has become irreversible. The treatment should be started when the grade of inflammation at the cellular level is moderate, at the beginning of the clinical signs, and before the destruction of neurons has been activated. During the inflammatory processes, as observed in neurodegenerative diseases, the development of GH resistance is one of the most important metabolic derangements found [166], as observed at hepatic level [167]. The inflammatory state exerts an inhibition on GH and IGF-1 bioactivity [168]. Pro-inflammatory cytokines reduce JAK2 and STAT activation through the stimulation of suppressor of cytokine signaling (SOCS) proteins, which are a central component of the regulation of GH and IGF-1 action [169] and inhibition of IGF-1 signaling pathway [170]. Pro-inflammatory cytokines inhibit the nuclear factor-κB (NF-κB), which play an important role in the regulation of GH/IGF-1 signaling [171], so that, during the neurodegenerative process, the effect of GH and IGF-1 therapy could be canceled by high cytokine expression at the cellular level. Neuroinflammation can be sustained by systemic inflammation and favors progress of the disease in the brain, as observed in AD [148].

Furthermore, other hormones mediate the effect of IGF-1 on tissues, usually not considered in these clinical trials. Thyroid hormones are also involved in the neurodegenerative process, as T3 has a stimulating effect on the SCG10 expression, providing the enhancement of peripheral nerve regeneration [172].

Sex steroids have a great interaction with GH/IGF-1 axis. During puberty, there is a strong synergistic interaction between estrogen and androgen with GH/IGF-1 action on body development [173]. Estrogen receptor concentrations (ER-α and ER-β) are highly expressed in adult female and male brains [174]. Androgen receptors have been found in the forebrain, midbrain, brain stem, and spinal cord [175], in hippocampal dendritic spines [176], and in axons of the cerebral cortex [177]. Androgens and estrogen can boost GH secretion [178]. In the male, testosterone induces an increase in GH secretion, and a consequent increase in IGF-1 plasma levels, while in women, estrogen induces an increase in GH secretion, followed by a decrease in IGF-1 [179]. In adult and children females, the response to GH is less evident than in male [180]. However, the effects of sex steroids on the GH/IGF-1 axis have been underestimated.

17β-Estradiol has shown a marked neuroprotective effect in the global cerebral ischemia model [181], and against stroke [182] in women and men, also [183]. Androgens have a significant effect on myelin regeneration [184], and have been proposed for peripheral nerve damage or brain trauma recovery in animals [185].

Gender has different effects on the incidence of ALS. In men, a greater incidence in younger age subjects with onset in the spinal regions was found, while in women, the onset was in the bulbar region [186]. Male to female ratio was 2.5 in the younger group, and 1.4 in older [187]. The incidence of AD is greater in women than in men, and seems correlated to the loss of protective effect of estrogen on mitochondria against β-amyloid toxicity in post-menopausal women [188]. In AD, sex hormones should be considered in a gender-specific program of prevention and treatment [189]. In Parkinson’s disease, the incidence rate men/women was 1.49, increasing with age, and sex hormone-related risk is suspected [190].

Estrogens modulate the effect of IGF-1, suppressing the serum level of IGF-BP3 in a dose-dependent manner, and the actions of oral estrogen are independent of endogenous GH status [191]. The activation of estrogen receptors mediates the neuronal survival by IGF-1 [192]. The androgen levels (dihydrotestosterone, and testosterone) influence the response to GH administration. Low levels of androgens are involved in a large number of neurodegenerative disorders [193,194]. Testosterone has an important neuroprotective effect [195], and modulates hippocampal structures, and functions [196] with a sexual differentiation manner, because in females, hippocampus neurogenesis and cell death is regulated by estrogen [197]. Androgens stimulate motoneurons and axons’ extension and regeneration [198,199]. The absence of testosterone markedly augmented oxidative alterations in nervous system tissues, especially in the hippocampus, evidencing that a low testosterone level predisposes the brain to oxidative injury [200]. Both sex hormones have a neuroprotective effect and are inversely related to AD risk [189]. In multiple sclerosis, the testosterone treatment has been proposed because of its property to increase the gray matter significantly in the frontal cortex [201]. On the other hand, testosterone, at high doses, determines the loss of dopamine neurons under oxidative stress conditions, as demonstrated by the increased incidence of Parkinson’s disease in men in comparison to women [202]. Importantly, androgens exert an anti-inflammatory effect, inhibiting cytokine release through androgen receptor activation [203,204], so the evaluation of clinical effects of GH or IGF-1 administration, without considering the serum level of sex hormones, can lead to erroneous results.

Finally, the IGF-1 level is influenced not only by GH secretion, but also by nutrition, and exercise [205]. Physical exercise is a determinant factor that stimulates significantly IGF-I, IGF-II, and IGFBP-3 serum levels, which are independent of GH secretion [206]. Hippocampal neurogenesis is strongly activated by running in human and animal models [207]. The beneficial effects of circulating levels of IGF-1 in the brain are potentiated by exercise [208].

GH and IGF-1 serum levels are increased in fit adolescent girls [209], and IGF-1 levels correlated with aerobic fitness and muscular endurance in healthy men [210]. In healthy older women, physical activity of moderate intensity increased IGF-1 levels [211]. Greater physical activity was associated with an increased brain volume and greater white matter integrity, evaluated before and after years of an exercise program in middle-aged adults [212]. Exercise enhanced IGF-I, and IGF-1 signaling in the ischemic brain can reduce brain ischemia [213]. Fitness condition, before a stroke, improved the recovery of function and neuroprotection [214]. However, intensive physical activity and low body mass index are strongly related to ALS [215]. Longitudinal studies have shown that a lower body mass index before the onset of the ALS is correlated with higher mortality [216,217]. A low body mass is correlated with undernutrition, evidencing that nutrition is also involved in the neurodegenerative process. The insulin/IGF-1 system is responsive to nutritional status, and during undernutrition, the brain IGF-1 expression is significantly reduced [218]. Ketogenic diet enhanced the IGF-1 receptor expression [219] and adequate caloric and macronutrient ingestion improves the effect of growth factor therapy [220]. A high-fat diet predisposes to neuroinflammation in central and peripheral nervous systems, and AD [221].

## 5. Conclusions

GH and IGF-1 play a fundamental role in growth and maintenance of CNS and PNS, and a positive effect on clinical outcome has been observed in TBI patients. However, lack of clinical studies in human subjects about the effects of exogenous administration of GH and IGF-1, and the evolution of neurodegenerative diseases, make it difficult to draw conclusions. The positive effects of IGF-1 therapy in ALS patients are limited, and probably due to the advanced state of the inflammatory process and pathologic alteration in neurons, so that their survival is severely compromised or irreversible. The level of severity of the disease can suggest that, when neurons are deeply damaged, functional recovery is impossible Treatment should be started as a preventive therapy in the first stages of neurological disease. The efficacy of GH/IGF-1 therapy is related to the plasma level of sex hormones, such as testosterone and estradiol. Furthermore, nutrition and physical activity also play an important role. However, against our conclusions, one of the major critical arguments is the longevity-related low IGF-1 levels found in nonagenarians [222], but of course, we do not know the central nervous activity of the IGF-1 receptors and all the other hormones possibly involved in neuroprotection. More specific clinical trials are necessary to evaluate the potential therapeutic effect of GH and IGF-1 on the brain, considering also sex hormones, nutrition, and physical exercise.

## Figures and Tables

**Table 1:** Effect of growth hormone (GH) therapy in patients who suffered traumatic brain injury.

Authors	Patients	Age	Type of Study	Duration	GH Dose (mg/day)	Clinical Outcome
Gardner, 2015 [82]	161	42.6	Clinical study	1 year	0.37 mg/day	GH therapy achieved clinically relevant, long-term benefit in quality of life
Moreau, 2013 [83]	23	37.9	Clinical trial	1 year	0.3 mg/day up to 0.6 mg/day	improve cognition and quality of life
Devesa, 2013 [90]	13	6–53	Follow-up	8 months	1 mg/day, 5 days/week, resting 15-days every 2-months	All patients improved during and at the end of treatment. Cognitive and motor improvements also swallowing. Visual performance ameliorated in amaurosis
Reimunde, 2011 [85]	11 M (mean 44.5 months after injury)	53.3	Clinical trial	3 months	0.5 mg/day for 20 days, then 1 mg/day for 5 days/week	Significative improvement of cognitive parameter, total IQ, and WAIS scale
High, 2010 [86]	23	39.1	Randomized Controlled Trial	1 year	0.2 mg/day, increasing 0.2/month up to 0.6 mg/day	Significant improvements of the cognitive impairments that are partially reversible
Maric, 2010 [87]	6 5 M 1 W	38.6	Follow-up	6 months	0.3 mg for males and 0.4 mg for female sc	Cognitive abilities, (particularly verbal and non-verbal memory) and psychiatric functioning were significantly improved
Kreitschmann-Andermahr, 2008 [88]	854 (28 childhood)	36.7	Follow-Up	1 year	0.3 mg (starting dose)	Improvement of quality of life
Hatton, 2006 [89]	49 GH/IGF-1 treated	30	Randomized double-blind study	14 days	IGF-1/GH therapy IGF-1 continuous intravenous infusion (0.01 mg/kg/h), and GH (0.05 mg/kg/day)	IGF-I and GH produced sustained improvement in metabolic and nutritional endpoints

1140 patients, mean age 41.0 ± 10.6; ALS functional rating scale-revised (ALSFRS-R). WAIS = Wechsler Adults Intelligence Scale IQ = intelligence quotient.

**Table 2:** Effect of rhIGF-1 and GH for the treatment of amyotrophic lateral sclerosis.

Authors	Patients	Age	Type of Study	Therapy	Doses of Therapy	Duration	Clinical Effects
Sorenson, 2008 [112]	110 M 57 W (placebo 163)	53.9	Clinical Trial	IGF-1	0.05 mg/kg body weight twice daily	2 years	Not provide benefit for patients with amyotrophic lateral sclerosis.
Nogano, 2005 [113]	5 M 4 W	46 49.5	Randomized Controlled Trial	IGF-1	Intrathecal administration high dose = 3 microg/kg body weight low dose = 0.5 microg/kg of body weight of IGF-1 every two weeks	40 weeks	High-dose treatment slowed a decline of motor functions of the ALS patients in total Norris and limb Norris scales, but not in bulbar Norris or vital capacity.
Borasio, 1998 [114]	183	51	Randomized Controlled Trial	IGF-1	0.1 mg/kg/day	9 months	Treatment showed no significant difference between groups and was safe and well tolerated.
Lai, 1997 [115]	266	52.5	Randomized Controlled Trial	IGF-1	0.05 mg/kg/day or 0.10 mg/kg/day	9 months	Slowed the progression of functional impairment and the decline in health-related quality of life. High and low doses had similar effects.
Smith, 1993 [109]	75 41 M + 34 W	57.1	Double-blind controlled trials	GH	0.1 mg/day three time/week	12–18 months	Survival analysis at 12 months did not reveal a difference between the treatment and placebo group. No change in IGF-1 plasma level.
Saccà [84]	45 24 M + 16 F	62.7	randomized, placebo-controlled, double-blind	GH + riluzole	0.6 mg (2 IU) s.c. every other day increased up to 2.4 mg (8 IU)	12 month	No effect on cerebral NAA or clinical improvement Note that IGF-BP3 was reduced after therapy.

Number patients included = 625, mean age 51 ± 2.9. M = men, W = women, NAA = *N*-acetylaspartate.
